# Characterization of aptamer-mediated gene delivery system for liver cancer therapy

**DOI:** 10.18632/oncotarget.23564

**Published:** 2017-12-21

**Authors:** Zhongbing Liu, Xiaoduan Sun, Shuangli Xiao, Yan Lin, Chunhong Li, Na Hao, Meiling Zhou, Ruolan Deng, Siyun Ke, Zhirong Zhong

**Affiliations:** ^1^ Department of Pharmaceutical Sciences, School of Pharmacy, Southwest Medical University, Luzhou, Sichuan 646000, China; ^2^ Department of Pharmacy, The Affiliated Hospital of Southwest Medical University, Luzhou, Sichuan 646000, China; ^3^ Luzhou Senior High School, Luzhou, Sichuan 646000, China

**Keywords:** liver cancer, adenovirus, PTEN, aptamer EpDT3, cell migration

## Abstract

Liver cancer is a fatal disease with limited therapy options. The recombinant adenovirus expressing tumor-suppressor gene of PTEN (Ad5-PTEN) showed effective antitumor activity against liver cancer. However, its disadvantages produced great limitation on its application, especially its nonspecific and toxicity to normal cells and tissues. The epithelial cell adhesion molecule (EpCAM) is over-expressed in some liver cancer cells and an RNA aptamer EpDT3 could specially target to EpCAM-positive cells. Based on this founding, we aimed to design a kind of gene delivery system of EpDT3-mediated Ad5-PTEN (EpDT3-PEG-Ad5-PTEN, EPAP) in which polyethylene glycol was used to be a linker to conjugate EpDT3 with Ad5-PTEN. This strategy may overcome the disadvantages of naked Ad5-PTEN and enhance the antitumor effect on liver cancer. The SDS-PAGE electrophoresis, TBE-PAGE electrophoresis and fluorescence detection were conducted to confirm the successful preparation of EPAP. Compared with the naked Ad5-PTEN, EPAP showed significant anti-proliferative and anti-migratory activities against HepG2 cells. EPAP also showed selective and precise target ability to EpCAM-positive HepG2 cells *in vivo*. Therefore, EPAP may be further explored as a novel effective anticancer drug for malignant liver cancer.

## INTRODUCTION

Liver cancer, a highly aggressive malignant tumor, is one of the major cancer killers. In the less developed countries, liver cancer among men is the second most frequently diagnosed cancer and it is the leading causes of cancer death [1]. Although surgical resection or liver transplantation is a curative treatment for hepatocellular carcinoma (HCC), most of these advanced patients are not candidates for surgery. Currently, the systemic chemotherapeutic treatment is in-effective against HCC, and in generally no single drug or drug combination could cure cancers due to its high metastasis and recurrence rate [2]. Furthermore, traditional chemotherapy generally caused severe cytotoxicity and side effect because the non-specific targeting and selectivity against cancer stem cells. Thus, it is urgent to find novel targeting therapeutic approaches to treat this disease.

The phosphatase and tension homolog (PTEN), a tumor suppressor gene [3], plays an important role in physiological activities, such as cell proliferation, migration and so on [4]. The mutation or inaction of PTEN was observed at high frequency in liver cancer and also was confirmed to be highly associated with hepatocellular carcinoma [5, 6]. But it needs a high efficient vector to transport PTEN to tumor cells and tissues. Some previous work showed that replication-deficient adenovirus is a promising gene delivery vector with high transfection efficiency, being easily produced in high titers, and no integration into host genome [7]. PTEN gene mediated by adenovirus type 5 (Ad5-PTEN) could effectively suppress the murine HCC [8]. Additionally, Epithelial cell adhesion molecule (EpCAM), a cancer stem cell surface marker, is an interesting target with over-expression in many human solid tumors [9]. But the target ability of adenovirus in gene therapy is limited due to several extracellular barriers, e.g. immune surveillance. Fortunately, Shigdar and the co-authors designed a kind of RNA aptamer EpDT3, which is the first aptamer specifically recognizing the cancer stem cell marker EpCAM with no immunogenicity or toxicity [10]. Moreover, liver cancer HepG2 cells is demonstrated to be a kind of EpCAM-overexpression cells [11]. Therefore, it is essential and feasible to develop a novel aptamer EpDT3-mediated delivery system for Ad5-PTEN to improve the therapeutic effects for HCC.

**Figure 1:**
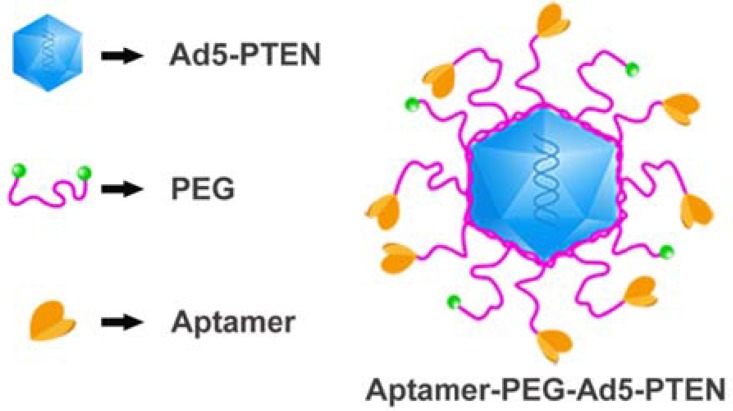
The schematic diagram of EpDT3-PEG-Ad5-PTEN (EPAP)

Therefore, we developed an RNA aptamer-mediated targeting drug delivery system with Ad5-PTEN to overcome the non-selectivity and non-specificity of Ad5-PTEN, reduce the toxicity against normal cells, and enhance the antitumor effect on human HCC. Firstly, we amplified and purified the recombinant adenovirus to get high titer and transfection efficiency of the recombinant adenovirus. Secondly, we conjugated EpDT3 with recombinant adenovirus Ad5-PTEN to form EpDT3-PEG-Ad5-PTEN (EPAP) (as shown in Figure 1) via chemical synthesis method, in which the RNA aptamer EpDT3 was connected with adenovirus by polyethylene glycol (PEG) through amide reaction principle. Then, we identified the formation of EPAP by electrophoresis method and fluorescence analysis. Furthermore, we assessed the various abilities of EPAP in cell proliferation inhibition, cell morphological change, and preventing cell migration *in vitro*. Finally, we investigated the uptake of EPAP by HepG2 tumor-bearing mice with intravenous administration *in vivo*.

## RESULTS

### Mass spectrometry result of EpDT3 and titer of Ad5

**Figure 2:**
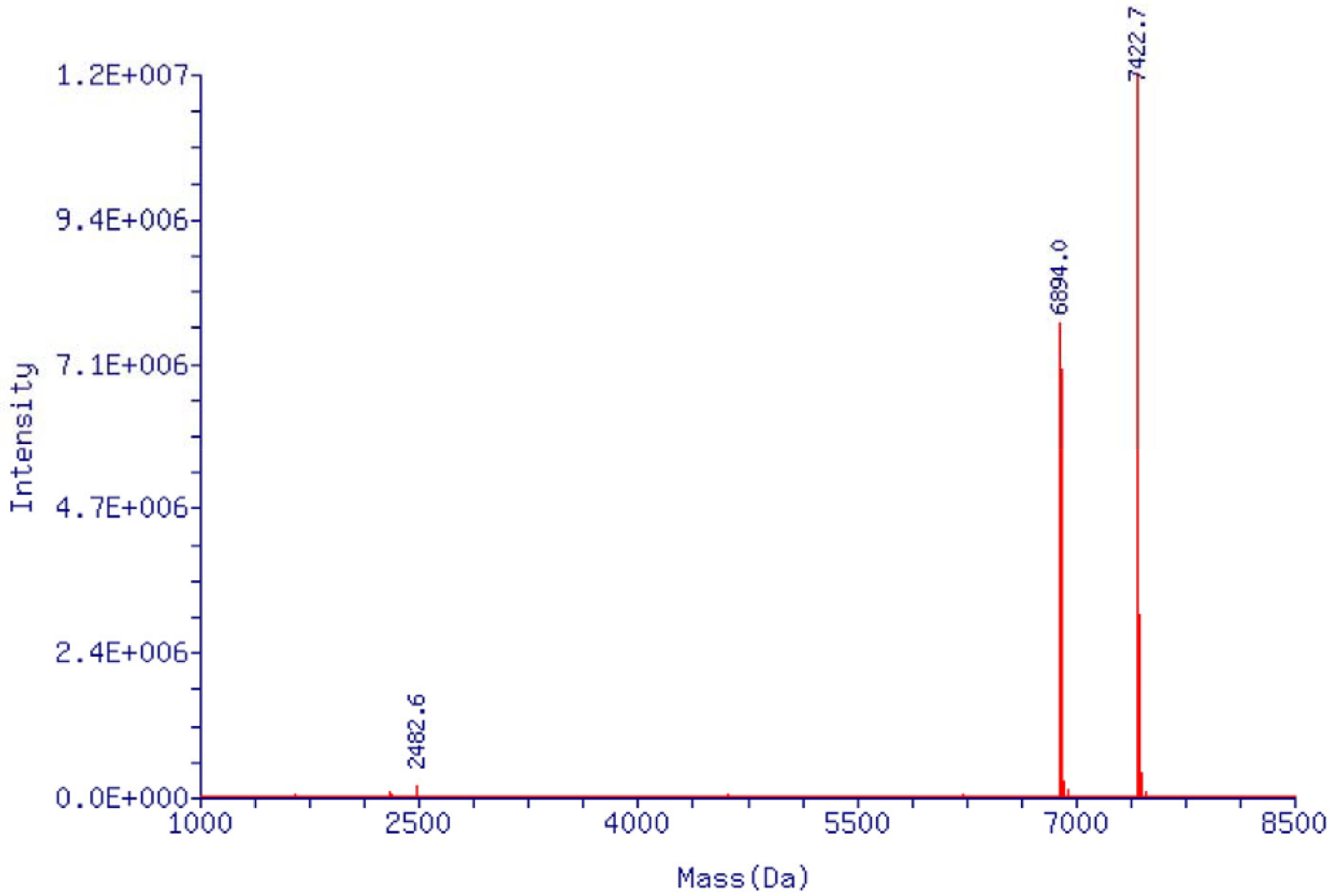
Mass spectrometry report of single stranded EpDT3 aptamer MS conditions were following as: probe (ESI), cone voltage (50 V), desolvation temp (350°C), capillary (3.00 KV), extractor (5 V), gas flow (3.5 L/min).

RNA aptamer EpDT3, with optimized sequence and simpler structure, is easy to be chemically synthesized and has high targeting efficiency to EpCAM-positive cancer cells. Therefore, in the current study we chose EpDT3 as the EpCAM-positive aptamer. It was purified by standard protocol and then identified by MS method. As revealed in Figure 2, the molecular weight of single strand RNA aptamer EpDT3 was ~7422 Dalton and the purified product was the EpDT3 aptamer what we need. Results from the CPE method and OD_260_ detection indicated that the titer of recombinant adenovirus was 4.32 × 10^9^ IFU/mL and 3.5 × 10^12^ VP/mL, respectively.

### Identification of PEG-Ad5-PTEN and EPAP

### Detection of PEG-Ad5-PTEN by SDS-PAGE

**Figure 3:**
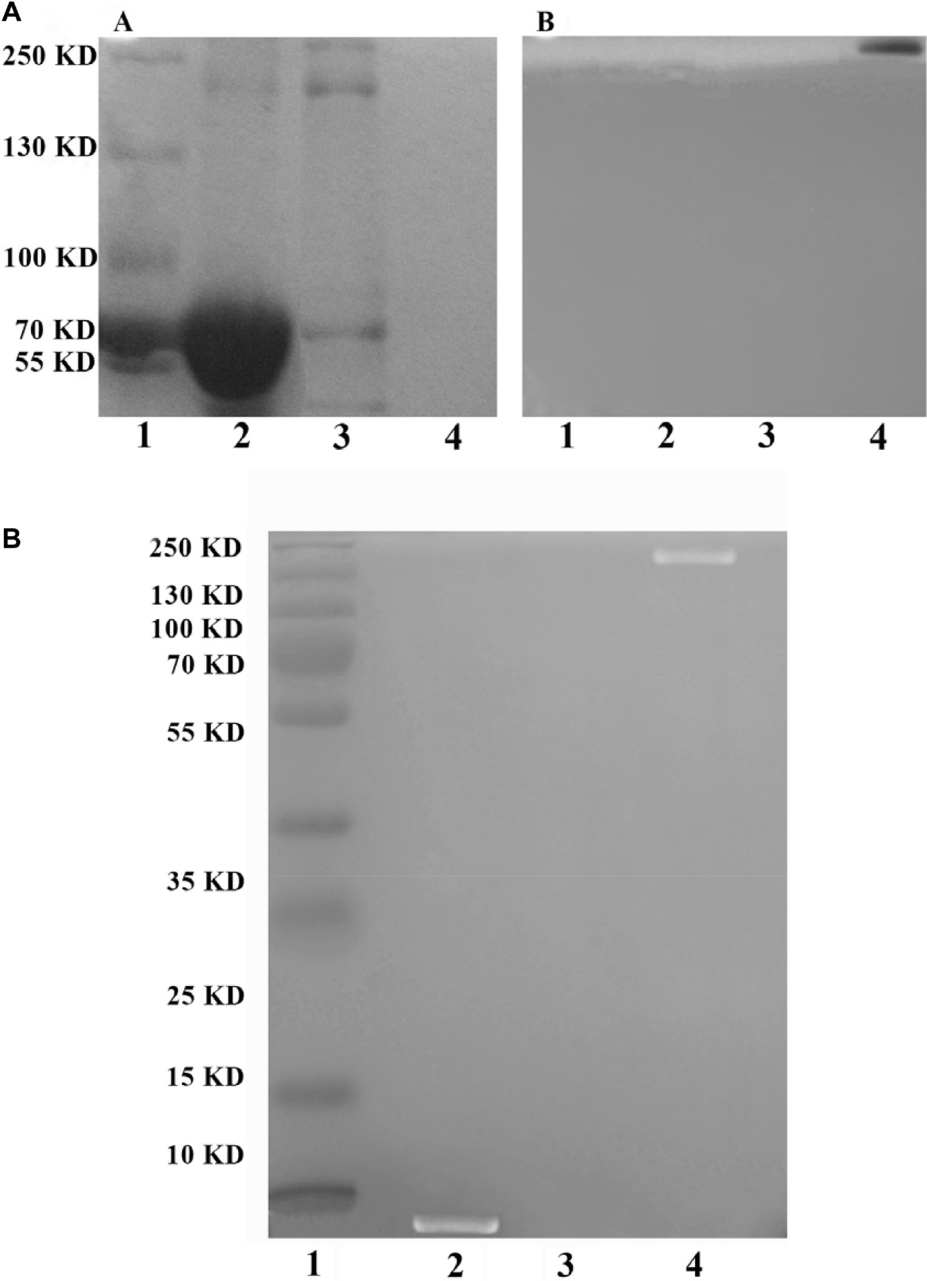
Detection of PEG-Ad5-PTEN by SDS-PAGE (**A**) and EPAP by TBE-PAGE (**B**). In Figure 3A, (A) It was stained by Coomassie Brilliant Blue; (B) It was stained by 0.1 mol/L iodine solution. Lane 1, marker; Lane 2, positive control protein of BSA; Lane 3, unmodified Ad5; Lane 4, PEG-Ad5-PTEN. In Figure 3B, it was stained by ethidium bromide. Lane 1, marker; Lane 2, EpDT3; Lane 3, PEG-Ad5-PTEN; Lane d 4, EPAP.

For SDS-PAGE, it is a fact that protein could be stained by Coomassie Brilliant Blue (CBB) but not by iodine, while PEG_2000_ (~2 kDa) could be dyed with 1% iodine but not by CBB. As shown in Figure 3A-A, marker (lane 1), BSA as a positive control (66 kDa, lane 2) and naked Ad5-PTEN (~ 200 kDa, lane 3) all showed the stained bands while PEG-Ad5-PTEN (lane 4) could not be displayed. Moreover, considering the molecular mass of Ad5-PTEN, it was obvious that the second band of lane 3 in Figure 3A-A was its band location, and the other two bands below might be the traces of the proteins from the purification process of Ad5-PTEN. In Figure 3A-B, only PEG-Ad5-PTEN exhibited one band while all the other three samples had no band on the gel. PEG-Ad5-PTEN in lane 4 showed an evident band in Figure 3A-B rather than in Figure 3A-A, which indicated no protein to be detected with CBB. Further, the PEG modification rate on capsid protein of Ad5-PTEN calculated by NIH ImageJ was 95.2 ± 4.58%. These results indicated that PEG was successfully connected to the most of Ad5-PTEN and the main product of reaction was almost all the complex of PEG-Ad5-PTEN.

### EPAP confirmation by TBE-PAGE

To confirm the formation of EPAP, a denaturing TBE-PAGE was performed. Based on the ethidium bromide staining method, only RNA aptamer EpDT3 and PEG-Ad5-PTEN modified with EpDT3 (EPAP, ~202 kDa) can be marked while PEG-Ad5-PTEN cannot be detected. From Figure 3B, there were bands in marker (lane 1), EpDT3 (~7 kDa, lane 2) and EPAP (~209 kDa, lane 4) lanes while PEG-Ad5-PTEN (~ 202 kDa, lane 3) could not be displayed. Furthermore, the band of EpDT3 was slightly lower than the band of 10 kDa while the band of EPAP was between the band of 250 kDa and 130 kDa in the standard marker. These results suggested that EPAP was prepared successfully. The yield of EPAP investigated using NIH ImageJ was 81.68 ± 2.95%.

### Fluorescence intensity detection of EPAP

Since the aptamer EpDT3 was modified by fluorescent substance of FAM in present study, the fluorescence intensity should be consistent with the concentration of EpDT3 or EPAP. Firstly, we set up the standard curve for EpDT3 aptamer by measuring the corresponding fluorescence intensities of a series concentration of EpDT3, and the results indicated that the concentration of EpDT3 were positively correlated with the fluorescence intensities from 1.9531 × 10^−8^ to 2.5 × 10^−6^ mol/L (Table 1). Therefore, the concentration range mentioned above were selected to establish the standard curve, and the linear equation was Y = 2 × 10^9^ X + 43.27 (R^2^ = 0.9998), in which X is the concentration of aptamer (mol/L) and Y is the fluorescence intensity.

**Table 1:** The relationship between concentration and fluorescence intensity of EpDT3 aptamer

Number	Concentration (mol/L)	Fluorescence intensity
A	2.50 × 10^−6^	5445.67 ± 132.54
B	1.25 × 10^−6^	2683.67 ± 74.86
C	6.25 × 10^−7^	1380.67 ± 15.18
D	3.13 × 10^−7^	726.57 ± 21.93
E	1.56 × 10^−7^	407.77 ± 57.18
F	7.81 × 10^−8^	237.60 ± 10.29
G	3.91× 10^−8^	109.29 ± 13.06
H	1.95 × 10^−8^	34.10 ± 10.03
I	9.78 × 10^−9^	21.35 ± 0.96

Results were expressed as the mean ± S.D. from three independent experiments.

**Figure 4:**
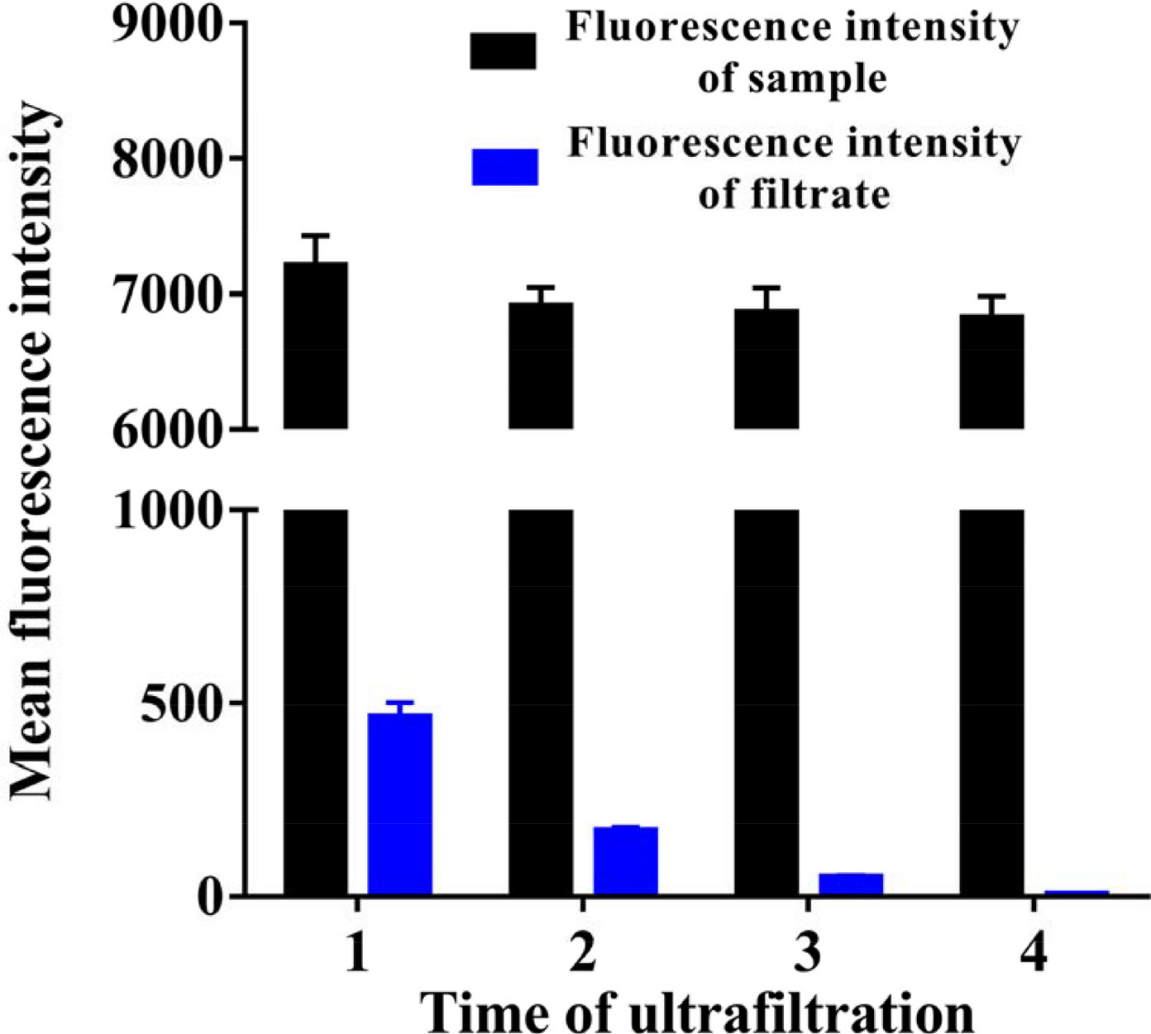
Comparison of fluorescence intensity of filtrate and samples before and after ultrafiltration

Furthermore, the variation of the fluorescence intensity in filtrate and the final fluorescence intensity of sample could be investigated to trace the free EpDT3 or EPAP. As shown in Figure 4 the fluorescence intensity of filtrate decreased gradually as the ultrafiltration running times increased. When the ultrafiltration centrifugation came up to the last time, the fluorescence intensity of filtrate became too weak to detect, which implied the disappearance of EpDT3. However, the fluorescence intensity of sample still remained 6823 ± 163.58 at the same time, which should result from EPAP. The yield of the final product of EPAP was calculated to be 90.44 ± 3.09%, which is higher than the result from TBE-PAGE electrophoresis. This phenomenon might be due to the fact that TBE-PAGE and fluorescence detection are based on different mechanisms and the sample preparation processes are also different.

The results from TBE-PAGE and fluorescence detection indicate that most of EpDT3 was successfully conjugated with COOH-PEG_2000_-Ad5-PTEN and EPAP was successfully prepared.

### Physical characterization of EPAP

As shown in Table 2, there was no significant increase in average particle size of EPAP (98.26 ± 2.85 nm) with a polydispersity index (PDI) of 0.254 ± 0.036, compared to that of naked Ad5-PTEN (92 ± 3.22 nm) with the PDI of 0.282 ± 0.024 (*P* > 0.05). However, the zeta potential of Ad5-PTEN changed from −10.17 ± 1.28 mV to −21.56 ± 9.75 mV after the formation of EPAP, which may be due to the contribution of aptamer EpDT3 with a zeta potential of −7.66 ± 3.51 mV. Interestingly, no changes were discovered for the zeta potential of EPAP during storage, which might suggest that the gene delivery system EPAP was dynamic stable.

**Table 2:** Size distribution and zeta potential of the naked Ad5-PTEN, EpDT3 and EPAP

	Size (nm)	Zeta potential (mv)	PDI
Ad5-PTEN	92.04 ± 3.22	−10.17 ± 1.28	0.282 ± 0.024
EpDT3	7.28 ± 0.36	−7.66 ± 3.51	0.246 ± 0.018
EPAP	98.26 ± 2.85	−21.56 ± 9.75	0.254 ± 0.036

PDI: polydispersity index. Results are expressed as mean ± SD from at least three measurements.

### EPAP leading to morphology change and growth inhibition for EpCAM-positive HepG2 cells

**Figure 5:**
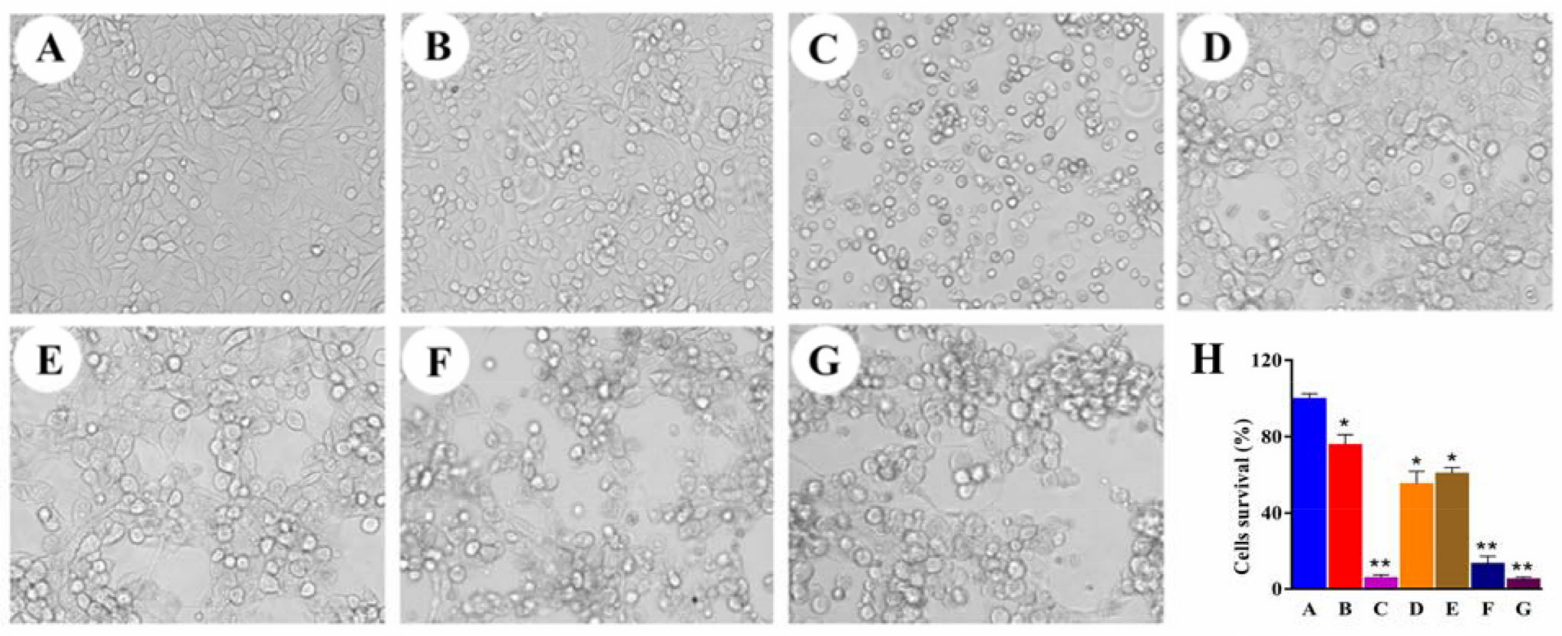
The cell morphological changes of HepG2 and quantitative analysis of the cell viabilities by MTT assay All cells were treated separately with various formulations as: (**A**) PBS, (**B**) EpDT3 (200 nM), (**C**) 5-FU (20 μg/mL), (**D**) Ad5-PTEN (MOI 40), (**E**) EpDT3-PEG-Ad5-PTEN (EPAP, MOI 20), (**F**) EPAP (MOI 40), (**G**) EPAP (MOI 80). Symbols represented statistical significance compared with the PBS control (^*^P < 0.05, ^**^P < 0.01).

To confirm the anticancer activity of EPAP against HepG2 cells, we observed the cell morphological change after treatment for 72 h. As shown in Figure 5A, HepG2 cells in PBS control group appear to be normal fusiform, bright color and clear boundary. The cells are branching, spreading on the surface of culture plate. In EpDT3 group (Figure 5B), the morphology alters slightly. The number of cells in other five different groups (Figure 5C–5G) are drastically reduced, the gap of cells became wider, and the lesions of cells become more severe in varying degrees, especially in 5-fluorouracil (5-FU, Figure 5C) and high-dose EPAP (Figure 5G) group. Moreover, the cell shapes also change to be round and irregular and most of cells after being treated with 5-FU, medium-dose EPAP (Figure 5F) and high-dose EPAP are dead. However, cells in the different concentrations of EPAP are similar and appear to be long fusiform at first and then become round and net-like gathered. The morphological changes of cells in three different concentrations of EPAP groups and 5-FU group are completely different in nature, which suggests that the mechanism of EPAP and 5-FU is certainly different. Additionally, the morphological changes with three different concentrations of EPAP are dose-dependent.

Additionally, we performed 3-(4, 5-dimethylthiazol-2-yl)-2,5-diphenyltetrazolium bromide (MTT) assay to quantitatively analyze the effect of EPAP on cell growth inhibition against HepG2 after 72 h treatment. Figure 5H indicated that the cell survival rates are 60.82 ± 3.01%, 13.47 ± 3.82%, and 5.29 ± 1.03% for HepG2 cells after treatment with EPAP at the concentrations of MOI 20, MOI 40 and MOI 80, respectively. However, the survival rats are 100 ± 2.55%, 75.89 ± 5.16%, 6.03 ± 1.39% and 55.34 ± 6.39% with the treatment of PBS, EpDT3 (200 nM), 5-FU (20 μg/mL) and Ad5-PTEN (MOI 40). Compared to the treatment group of Ad5-PTEN (MOI 40), both EPAP (MOI 40) and EPAP (MOI 80) showed significant anti-proliferative activities against HepG2 cells with ^**^P < 0.01. This result was consistent with that from the cell morphological change observation.

### EPAP reducing migration of HepG2 cells

**Figure 6:**
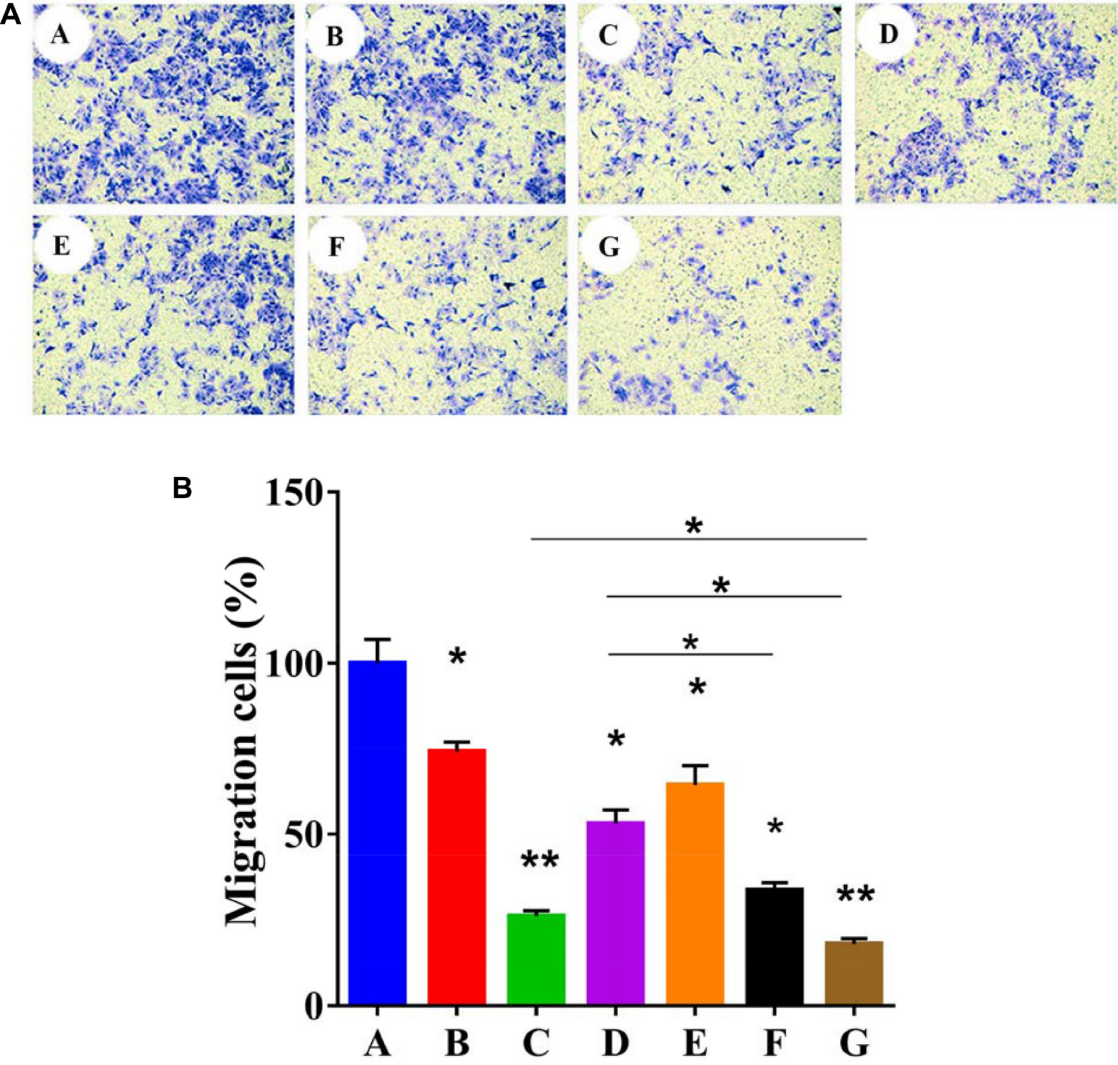
Qualitative (**A**) and quantitative (**B**) analyses of the *in vitro* cell-migration inhibition detection against HepG2 cells. All the tested cells were treated separately with different formulation as: (A) PBS, (B) EpDT3 (200 nM), (**C**) 5-FU (20 μg/mL), (**D**) Ad5-PTEN (MOI 40), (**E**) EPAP ((MOI 20)), (**F**) EPAP (MOI 40), (**G**) EPAP (MOI 80). Symbols represented statistical significance compared with the control group (^*^P < 0.05, ^**^P < 0.01).

In early reports, it was demonstrated that PTEN could inhibit the cell spreading and migration of tumor cells [12]. As shown in Figure 6A and 6B, compared with PBS group at 24 h post-administration, the relative migration rate of cells treated with EpDT3, 5-FU (20 μg/mL), Ad5-PTEN (40 MOI), EPAP (20 MOI), EPAP (20 MOI), EPAP (80 MOI) was 74.28 ± 2.57%, 26.13 ± 1.56%, 53.08 ± 4.05%, 64.40 ± 5.74%, 33.74 ± 2.17% and 17.9 ± 1.63%, respectively. These results were consistent with the results from the previous wound healing assay, and also demonstrated that EPAP obviously enhanced migratory inhibition against HepG2 cells compared to Ad5-PTEN, which implied that aptamer EpDT3 could enhance the suppression effect of Ad5-PTEN on HCC cell migration. Moreover, EPAP reduced the migratory activity of HepG2 cells in dose-dependent manner.

### Special uptake of EPAP by HepG2 xenograft tumor

**Figure 7:**
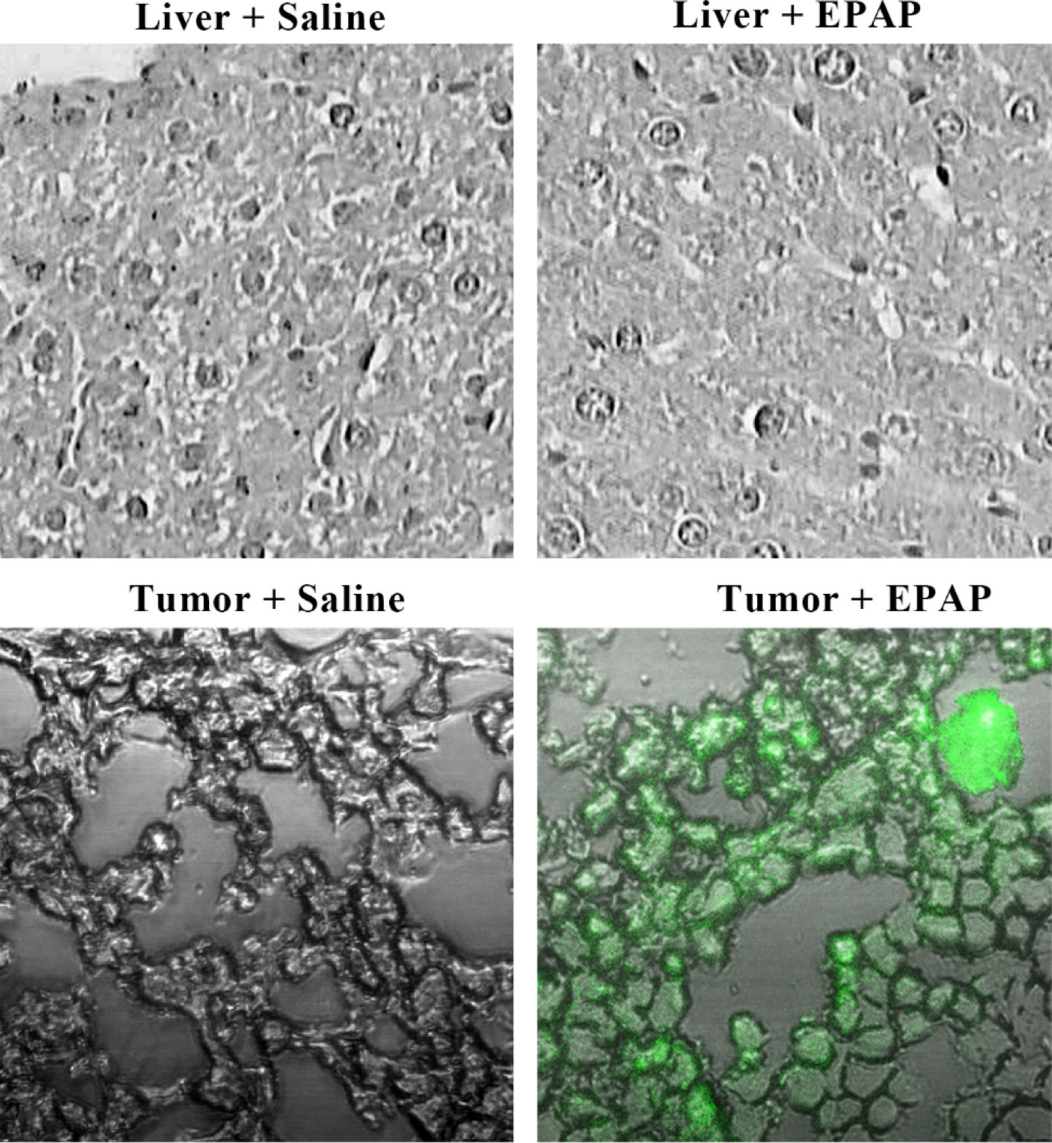
Uptake of EPAP by normal liver tissues and the HepG2 tumors treated by a single tail intravenous injection separately with 200 mL of saline as control and EPAP (4.32 × 10^9^ PFU/mL), respectively EPAP was labeled with FAM (green).

EPAP was labeled with FAM fluorescence (green) to study its distribution in tissues by fluorescence microscope. Figure 7 showed representative images of frozen sections of normal liver and tumors tissues under the laser scanning confocal microscope. After 24 h treatment, no green fluorescence was detected in both normal liver and tumor sections in saline group. However, when it is treated with EPAP, strong fluorescence was detected in tumor section while no fluorescence was shown in normal liver section. These results implied that EPAP could specifically target to EpCAM-positive HepG2 xenograft tumor but not to normal liver tissue.

## DISCUSSION

In the present study, through different methods we confirmed that EPAP was prepared successfully and that EPAP could significantly change the morphology of HepG2 cells and kill them, enhance the inhibition of migration activity of EpCAM-positive HepG2 cells *in vitro*. Ad5 vectors in EPAP also showed dose-dependent manners, which was in accordance with the previous investigation [13]. Moreover, EPAP showed good target ability to HepG2 tumor *in vivo* but not to normal liver tissues. Interestingly, EpDT3 alone induced some cellular morphology change and killing effects, and exhibited some anti-migratory activity against EpCAM-positive HepG2 cells *in vitro*. All these findings above have important significance in cancer therapy as EPAP may bear a strong power to suppress the progression and metastasis of human hepatocellular carcinoma. EpDT3 might provide another effective target and diagnostic approach for liver cancer.

During the PEGylation of Ad5, it was possible that the virus would react with both ends of COOH-PEG_2000_-COOH, which might lead to another product Ad5-PEG-Ad5 besides Ad5-PEG-COOH. To avoid this kind of embarrassing, we strictly controlled the ratio of the reagents in the reaction. Moreover, the results from SDS-PAGE electrophoresis also demonstrated that the main product was Ad5-PEG-COOH. Meanwhile, the EPAP complex was identified by TBE-PAGE electrophoresis and the fluorescence intensity detection. Our results manifested again that fluorescence detection method is a simple, acute and efficient detection method used in other reports [14, 15]. The results from the growth and migration inhibition tests showed that compared to naked Ad5-PTEN (MOI 40), the treatment of EPAP (MOI 40) could obvious inhibit the growth and migration of HepG2 cells. This demonstrated the difference between Ad5-PTEN and EPAP, which might suggest the formation of EPAP. Meanwhile, in the preparation process the crude products were centrifuged with ultrafiltration centrifugation to remove the free FAM-labeled EpDT3 and other small molecules. Since there is no free EpDT3 molecule, the green fluorescence in the targeted abilities assay *in vivo* might result from the EpDT3 conjugated with Ad5-PTEN, which may also suggest the formation of EPAP.

Further studies should be performed to detect the pharmacokinetics and mechanism of action of EPAP. We can also investigate its antitumor ability against other cancer cells. Moreover, a new dosage of EPAP may be developed to more greatly enhance the anticancer efficacy and to remove the potential side toxicity. Shigdar et al. reported EpDT3 is internalized into the cell via endocytosis after binding to EpCAM on the surface [10]. Therefore, this mechanism can be used to channel diverse substances including chemotherapeutic drugs. However, Hsu et al. reported that PTEN-overexpression cell lines are sensitive to chemotherapy with 5-FU [16]. In future work, the combined application of EPAP with 5-FU maybe has a promise to be a powerful approach in live cancer treatment.

## MATERIALS AND METHODS

### Materials

The FAM-labeled aptamer EpDT3 was got from Shanghai GenePharma Bio-technology Company (Shanghai, China). Recombinant adenovirus expressing the tumor-suppressor gene PTEN (Ad5-PTEN) or reporter gene LacZ (Ad5-LacZ) was obtained from VGTC Gene Technology (Beijing, China). The COOH-PEG_2000_-COOH (MW 2057) was purchased got from JENKEM Technology Corporation (Beijing, China). Protein marker was obtained from Thermo Fisher Scientific (Vilnius, Lithuania, EU). DMEM high glucose complete medium and RPMI 1640 medium were from Gibco Life Technologies Corporation (Grand Island, NY). 5-fluorouracil (5-FU) injection was provided by Tianjing Kingyork Group Company (Tianjing, China). 3-(4, 5-dimethylthiazol-2-yl)-2,5-diphenyltetrazolium bromide (MTT) was purchased from Biosharp Biotechnology Company (Beijing, China). All other reagents were purchased from Keyang Corporation (Luzhou, China).

### Cells and animals

The human hepatocellular carcinoma cells HepG2 and human embryonic kidney HEK293 cells were purchased from Shanghai Cell Institute, China Academy of Sciences. HepG2 and HEK293 cells were maintained in DMEM with 10% FBS and 100 units/mL of penicillin / streptomycin sulfates.

Blab/c female nude mice (18–22 g), 6-8 weeks of age, were got from Chengdu Dossy Experimental Animals Company (Chengdu, Sichuan, China, Certificate No. SCXK2015-030). The mice were acclimated for one week before being used in the study. During the experiment period, the mice were kept five mice/cage with water and food *ad libitum* under specific pathogen-free conditions according to an institutionally approved animal protocol (Permit No. 20160141).

### Design and synthesis of EpDT3

The RNA aptamer EpDT3 was modified with an amino in its 3′-end, namely “5′- GCGACU GGUUACCCGGUCG-(CH_2_)_6_-NH_2_-3′ (with 2′-fluro-modified ribose on all pyrimidines)”, and the 5’-end position was labeled with carboxyfluorescein FAM. It was synthesized by a solid phase chemistry method, purified by HPLC and identified by MS (HP1100LC/MS, Agilent Technologies, America).

### Preparation of recombinant adenovirus vectors and EPAP

The recombinant adenovirus Ad5 was amplified in HEK 293 cells. Infected cells were harvested once all the cells were infected by the virus and concentrated by centrifugation at 2200 × g at 4°C for 10 min. The virus vectors were purified by two ultracentrifugation steps in cesium chloride gradients and desalted as previously described [17]. Virus stocks were aliquoted and frozen at −80°C. The measurement of the virus titer was conducted by both cytopathic effect (CPE) method and OD260 detection method as previously mentioned [14, 18]. EPAP and EpDT3-PEG-Ad5-LacZ (EPAL) were prepared by conjugating EpDT3 with Ad5 through PEG linker via amide bond according to our previous study [19].

### Confirmation of PEG-Ad5-PTEN and EPAP

We conducted the sodium dodecyl sulfate-polyacrylamide gel electrophoresis (SDS-PAGE) and Tris/boric acid/EDTA polyacrylamide gel electrophoresis (TBE-PAGE) to confirm the formation of PEG-Ad5 and EPAP, respectively. Thereafter, the modified rate of capsid protein on PEG-Ad5 was analyzed and calculated using the image analysis software NIH ImageJ. The identification of PEG-Ad5-PTEN was performed by SDS-PAGE.

The SDS-PAGE experiment was performed as described in previous report [15]. Briefly, the electrophoresis buffer (1×, pH 8.3) including 25 mM Tris, 250 mM glycine and 3.5 mM SDS were prepared. Two identical PAGE gels were prepared with the concentrations of separation gel and spacer gel at 6% and 5%, respectively. Before loading, all samples including PEG-Ad5, Ad5 and BSA protein as a positive control were mixed with loading buffer (4×) at 3:1 (v/v) for 5 min at 95°C. The samples were loaded parallelly on the two identical separate gels and the electrophoresis was performed at a constant voltage of 120 V for 2.5 h. After electrophoresis, the two gels were stained respectively with Coomassie Brilliant Blue and iodine solution (0.1 M) overnight and decolorized until the background became transparent.

For TBE-PAGE detection, TBE buffer (89 mM Tris, 89 mM boric acid and 2 mM EDTA, pH 8.3) and PAGE gel with a concentration of 20% were prepared. All the samples including protein marker, EpDT3, PEG-Ad5 and EPAP were mixed with loading buffer at 10:1 (v/v) for 5 min at 95°C and loaded immediately after cooling on ice. After 30 min for pre-electrophoresis, about 10 μg of all samples were loaded orderly on the gel and the electrophoresis was performed at a constant voltage of 80 V for 5 h. After the electrophoresis, the gels were stained with ethidium bromide (5 mg/mL, Biotium) for 10 min followed by washing 3 times with TBE buffer. The exact location of all bands on the gel was identified and imaged using a gel documentation system under UV illumination.

Moreover, in the synthesis of EPAP we took the ultrafiltration to remove the non-reacted fluorescence-labeled EpDT3. Consequently, to further testify the formation of EPAP and judge whether the free small molecules EpDT3 has been removed thoroughly, we detected the fluorescence intensity of the external liquid and the internal samples of mixed solution with the same ten parts of the crude EPAP (Ex = 494 nm, Em = 518 nm) after ultrafiltration by an F-7000 fluorescence spectrophotometer (Hitachi, Tokyo, Japan) at the specified times. It was conducted in triplicate.

Additionally, we prepared the fluorescent standard curve for EpDT3 to quantify the product yield of EPAP. Firstly, RNA aptamer EpDT3 was dissolved in RNase-free water to get an original standard solution at a concentration of 2.5 × 10^−6^ mol/L. After diluted proportionally, the fluorescence intensities of all standard solutions were measured (Ex = 494 nm, Em = 518 nm) by fluorescence spectrophotometer to draw the standard curve of EpDT3 aptamer. Finally, the concentration of EPAP was obtained via the fluorescence value of EPAP and the standard curve of EpDT3 aptamer.

### Particle size and zeta potential detection

We investigated the mean particle size and the zeta potential of complexes by photon correlation spectroscopy (PCS) (NanoBrook 90Plus Zeta, Brookhaven Instruments Ltd., USA.). Before the determinations, all samples were diluted at a ratio of 1:2 (V/V) in RNase-free water. The measurements were carried out at a constant angle of 90 at normal temperature. It was analyzed in triplicate for 3 min every time (*n* = 3).

### Cell morphology observation and MTT assay

To study the anticancer efficacy of EPAP on HepG2 cells and further subjected to MTT assay. The cells in logarithmic growth phase were seeded onto 96-well plates at a density of 5×10^3^ cells per well. Until reached 70-80 % confluence, the cells were respectively treated for 4 h with 100 μL different formulations of saline as control, EpDT3 (200 nM), 5-FU (20 μg/mL) as positive control, naked Ad5-PTEN (MOI 40), low-dose EPAP (MOI 20), medium-dose EPAP (MOI 40), high-dose EPAP (MOI 80). The cells were further incubated at 37°C for 72 h. The morphologic changes of HepG2 cells were monitored under a light inverted microscope and photographed at 20 × magnification. Further, the cell viability was measured according to protocol described previously, and it was calculated according to the formula as: cell viability (%) = (*A*_treatment_ – *A*_background_) / (*A*_control_ – *A*_background_) × 100, in which the control cells were not subjected to treatment and the background well had no cells in it.

### Transwell migration analysis

Transwell migration assays were aimed to further investigate the migratory ability of HepG2 cells. It was performed in 24-transwell insert plates with 8-μm pore size (BD Biosciences, San Jose, USA) [20]. Approximately 0.1 mL of cell suspension at a density of 1.16 × 10^6^ cells/well was added to each top chamber. About 100 μL of different test compounds mentioned in cell morphology assay, prepared in starvation medium, were added to each upper chamber. Then each bottom chamber was filled with 0.5 mL of DMEM with 20% FBS as chemotactic agent. To count migrated cells more precisely, after incubated at 37°C with 5% CO_2_ for 24 h, the cells of the insert membranes were washed with PBS for three times and the non-migrated cells on the upper surface membrane were wiped out carefully by swabs. The migrated cells were fixed with 4% formaldehyde for 25 min, washed with PBS for three times, stained using 0.1% crystal violet for 10 min and washed fully with pure water. Finally, the mean number of migrated cells per field were calculated in five random different fields under an inverted microscope and photographed at 200× magnification.

### *In vivo* >tumor targeting assay

For detection of the target ability of EPAP *in vivo*, fluorophore-labeled EpDT3 and Ad5 were used to prepare the EPAP by the methods mentioned above. About 0.2 mL logarithmic human hepatocellular carcinoma HepG2 cells, 5 × 10^7^ cells / mL re-suspended in phosphate buffer saline (PBS), were subcutaneously inoculated into the right flank of BALB/c nude mice to form xenograft. Once the volume of the tumor reached ~300 mm^3^, the tumor-bearing BALB/c mice (three per group) were singe injected in the tail vein with 0.2 mL of either saline and EPAP (4.32 **×** 10^9^ PFU/mL), respectively. After treatment for 24 h, all animals were euthanized and the normal liver and tumor tissues were collected. Then these fresh tissues were immediately made into frozen sections (8 μm) separately and the green fluorescence was detected and imaged under laser confocal microscopy with 400 **×** magnification [21].

### Statistical analysis

Data from at least three independent experiments are shown as mean ± SD, unless otherwise stated. The differences between two groups were analyzed by one-way analysis of variances or Student's *t*-test using the SPSS statistical software 17.0 (SPSS, Chicago, IL). *P* < 0.05 means statistically significant and *P* < 0.01 indicates very significant.

We thank for getting help and supports from the following research platforms of the Collaborative Innovation Center for Prevention and Treatment of Cardiovascular Disease, Southwest Medical University, Luzhou, Sichuan 646000, China.

## References

[1] Torre LA, Bray F, Siegel RL, Ferlay J, Lortet-Tieulent J, Jemal A (2015). Global cancer statistics, 2012. CA Cancer J Clin.

[2] Llovet JM, Bruix J (2003). Systematic review of randomized trials for unresectable hepatocellular carcinoma: Chemoembolization improves survival. Hepatology.

[3] Li J, Yen C, Liaw D, Podsypanina K, Bose S, Wang SI, Puc J, Miliaresis C, Rodgers L, McCombie R, Bigner SH, Giovanella BC, Ittmann M (1997). PTEN, a putative protein tyrosine phosphatase gene mutated in human brain, breast, and prostate cancer. Science.

[4] Shearn CT, Petersen DR (2015). Understanding the tumor suppressor PTEN in chronic alcoholism and hepatocellular carcinoma. Adv Exp Med Biol.

[5] Yamazaki K, Eng C, Kuznetsov SA, Reinisch J, Yamashita DD, Walker J, Cheung C, Robey PG, Yen SL (2015). Missense mutation in the PTEN promoter of a patient with hemifacial hyperplasia. Bonekey Rep.

[6] Sendor AB, Hacker KE, Chen S, Corona AL, Sen O, Chiang DY, Snavely A, Rogers AB, Montgomery SA, Rathmell WK, McRee AJ (2015). Von Hippel-Lindau status influences phenotype of liver cancers arising from PTEN loss. Gastrointest Cancer.

[7] Zhong ZR, Shi SJ, Han JF, Zhang ZR, Sun X (2010). Anionic liposomes increase the efficiency of adenovirus-mediated gene transfer to coxsackie-adenovirus receptor deficient cells. Mol Pharm.

[8] Liu Z, Li J, Li J, Huang J, Ke F, Qi Q, Jiang X, Zhong Z (2012). Mannan-modified Ad5-PTEN treatment combined with docetaxel improves the therapeutic effect in H22 tumor-bearing mice. Int J Nanomedicine.

[9] Visvader JE, Lindeman GJ (2008). Cancer stem cells in solid tumours: accumulating evidence and unresolved questions. Nat Rev Cancer.

[10] Shigdar S, Lin J, Yu Y, Pastuovic M, Wei M, Duan W (2011). RNA aptamer against a cancer stem cell marker epithelial cell adhesion molecule. Cancer Sci.

[11] Zhao X, Parpart S, Takai A, Roessler S, Budhu A, Yu Z, Blank M, Zhang YE, Jia HL, Ye QH, Qin LX, Tang ZY, Thorgeirsson SS (2015). Integrative genomics identifies YY1AP1 as an oncogenic driver in EpCAM(+) AFP(+) hepatocellular carcinoma. Oncogene.

[12] Stewart AL, Mhashilkar AM, Yang XH, Ekmekcioglu S, Saito Y, Sieger K, Schrock R, Onishi E, Swanson X, Mumm JB, Zumstein L, Watson GJ, Snary D (2002). PI3 kinase blockade by Ad-PTEN inhibits invasion and induces apoptosis in RGP and metastatic melanoma cells. Mol Med.

[13] Zhong ZR, Wan Y, Han JF, Shi SJ, Zhang ZR, Sun X (2011). Improvement of adenoviral vector-mediated gene transfer to airway epithelia by folate-modified anionic liposomes. Int J Nanomedicine.

[14] Mittereder N, March KL, Trapnell BC (1996). Evaluation of the concentration and bioactivity of adenovirus vectors for gene therapy. J Virol.

[15] Jing P, Cao S, Xiao S, Zhang X, Ke S, Ke F, Yu X, Wang L, Wang S, Luo Y, Zhong Z (2016). Enhanced growth inhibition of prostate cancer *in vitro* and *in vivo* by a recombinant adenovirus-mediated dual-aptamer modified drug delivery system. Cancer Lett.

[16] Hsu CP, Kao TY, Chang WL, Nieh S, Wang HL, Chung YC (2011). Clinical significance of tumor suppressor PTEN in colorectal carcinoma. Eur J Surg. Oncol.

[17] Xiong D, Liu Z, Bian T, Li J, Huang W, Jing P, Liu L, Wang Y, Zhong Z (2015). GX1-mediated anionic liposomes carrying adenoviral vectors for enhanced inhibition of gastric cancer vascular endothelial cells. Int J Pharm.

[18] Nyberg-Hoffman C, Shabram P, Li W, Giroux D, Aguilar-Cordova E (1997). Sensitivity and reproducibility in adenoviral infectious titer determination. Nat Med.

[19] Xiao S, Liu Z, Deng R, Li C, Fu S, Chen G, Zhang X, Ke F, Ke S, Yu X, Wang S, Zhong Z (2017). Aptamer-mediated gene therapy enhanced antitumor activity against human hepatocellular carcinoma *in vitro* and *in vivo*. J Control Release.

[20] Fang KP, Zhang JL, Ren YH, Qian YB (2014). Talin-1 correlates with reduced invasion and migration in human hepatocellular carcinoma cells. Asian Pacific journal of cancer prevention. APJCP.

[21] Papahadjopoulos D, Allen TM, Gabizon A, Mayhew E, Matthay K, Huang SK, Lee KD, Woodle MC, Lasic DD, Redemann C, Martin FJ (1991). Sterically stabilized liposomes: improvements in pharmacokinetics and antitumor therapeutic efficacy. PNAS.

